# Targeting SNHG3/miR-186-5p reverses the increased m6A level caused by platinum treatment through regulating METTL3 in esophageal cancer

**DOI:** 10.1186/s12935-021-01747-9

**Published:** 2021-02-17

**Authors:** Mingxin Zhang, Minghua Bai, Li Wang, Ning Lu, Jia Wang, Rong Yan, Manli Cui, Honglin Yan, Lingmin Zhang

**Affiliations:** 1 Department of Gastroenterology, The First Affiliated Hospital of Xi’an Medical University, No. 48 Feng Hao West Road, Xi’an, 710077 Shaanxi China; 2 Department of Health, Liaocheng People’s Hospital, Liaocheng, 252000 Shandong China; 3 Department of Scientific Research, The First Affiliated Hospital of Xi’an Medical University, Xi’an, Shaanxi China; 4 Department of Anesthesiology, The First Affiliated Hospital of Xi’an Jiaotong University, No. 277 Yanta West Road, Xi’an, 710061 Shaanxi China

**Keywords:** m6A level, METTL3, SNHG3, miR-186-5p, Platinum

## Abstract

**Background:**

Platinum-based chemotherapy is a mainstay for treating esophageal cancer patients. In this manuscript, we have provided clues for influence of platinum on overall m6A level and further investigated the potential regulatory mechanism.

**Methods:**

qRT-PCR was used to measure SNHG3 and miR-186-5p expression; ELISA and western blot were used to measure the expression of METTL3. CCK8 was used to measure the cell proliferation rate. Caspase 3/7 activity was used to measure the apoptosis rate. Dual luciferase reporter gene assay and RNA pull down assay were used to investigate the potential crosstalk between miR-186-5p and SNHG3; and miR-186-5p and METTL3.

**Results:**

m6A level was increased when treated with platinum (CDDP, CPB and L-OHP). Besides, SNHG3 expression was induced and miR-186-5p expression was suppressed by platinum. Furthermore, SNHG3 could promote the m6A level, however miR-186-5p inhibited the m6A level through targeting METTL3. SNHG3 interacts with miR-186-5p to negatively regulate the expression of miR-186-5p; and miR-186-5p might bind to the 3′UTR of METTL3 to regulate its expression.

**Conclusion:**

Platinum can increase the overall m6A level of esophageal cancer. SNHG3/miR-186-5p, induced by platinum, was involved in regulating m6A level by targeting METTL3. Our manuscript has provided clues that regulating m6A level might be a novel way to enhance the platinum efficacy.

## Introduction

Esophageal carcinoma is one of the most common malignant tumors. It ranks the seventh in incidence and sixth in mortality all over the world [1]. In China, esophageal carcinoma is the leading cause of cancer death [2]. Esophageal cancer is mainly composed of two pathological types (adenocarcinoma and squamous cell carcinoma), which have significant different etiologies and treatment strategies.

Platinum-based drug plus 5-fluorouracil (FP) is the first line regimen for esophageal squamous cell carcinoma, especially for patients at advanced stages. It was reported that paclitaxel plus lobaplatin showed satisfying therapeutic efficiency and less toxicity in ESCC patients [3]. Platinum-based drugs include carboplatin, cisplatin and oxaliplatin [4], which target the DNA to interfere the cell cycle progression and replication, by forming adducts to DNA strand crosslinks [4]. Although platinum-based agents are widely applied in the clinical treatment in ESCC, platinum resistance frequently leads to local recurrence and poor prognoses of patients. Therefore, it is urgent to explore the specific mechanisms of platinum resistance in ESCC.

N6-methyladenosine (m6A) is one of the most abundant RNA modification types in mammals [5]. m6A is induced by the methyltransferase complex, including METTL3–METTL14 and erased by demethylases, including ALKBH5 and FTO [6]. Accumulating evidences demonstrated that m6A mediates important functions in cancer progression and chemo-radio therapy resistance [7]. For example, Yan et al. reported that cells with hypomethylation of mRNA m^6^A were more resistant to tyrosine kinase inhibitor (TKI) therapy [8]. Zhou et al. found that FTO expression was upregulated in cervical squamous cell carcinoma to reduce m6A level, and finally leading to chemotherapy and radiotherapy resistance [9]. Shriwas et al. found that Human RNA helicase, DDX3, promoted cisplatin resistance of ESCC by directly regulating m^6^A demethylase ALKBH5 and decreased m^6^A methylation level in FOXM1 and NANOG [10]. Here, we found that CDDP, CPB and L-OHP could enhance the m6A level in ESCC cells. And we hypothesized that enhanced m6A level might result in platinum resistance.

Accumulating evidence has shown that LncRNAs plays important roles in regulating various biological functions of cancers, including chemotherapy resistance [11]. SNHG3 was previously found to exert oncogenic roles in a plethora of cancers: Based on current studies, SNHG3 was involved in TGF-β, NOTCH, JAK2/STAT3 and HGF pathway to promote cancer cell proliferation, invasion and inhibit apoptosis rate [12–14]. Besides, Xuan et al. has presented evidences that SNHG3 interacts with EZH2 to regulate the methylation status of MED18 and finally suppress MED18 expression, leading to gastric cancer progression [15]. However, the specific role of SNHG3 in esophageal cancer was not reported yet. In this manuscript, we assumed that SNHG3 was essential in mediating m6A level and platinum resistance by regulating METTL3.

## Method

### Patients

348 esophageal cancer patients were recruited from 2013 to 2015 in First affiliated hospital of Xi’an medical University. All patients were pathologically diagnosed after esophagectomy. All patients were informed of cancer and normal esophageal tissues and signed the consent. This study meets the standard of Helsinki declaration and is approved by First affiliated hospital of Xi’an jiaotong University and Xi’an medical University.

### Cell culture and transfection

KYSE-150 and Eca-9706 were purchased from Cell Bank of Chinese Academy of Sciences (Shanghai, China). RPMI 1640 supplemented with 10% fetal bovine serum (Gibco, USA) were used for cell culture. The culture condition is 5% CO_2_ and 37 °C. Cells were transfected with SNHG3 down regulated lentivirus, METTL3 and miR-186-5p up and down regulated lentivirus, which were purchased from Genechem (Shanghai, China) according to the manufacturer’s instructions.

### ELISA

SimpleStep ELISA® (ab270552, Abcam, Shanghai, China) was purchased to perform ELISA tests. Around 1 × 10^6^ cells were used to resuspend at 3 mL medium; 50 μL cell lysates were added at each well for evaluation. 50μL METTL3 antibody (1:1000, Invitrogen, Shanghai, China) Then 100 μL detection reagent was added and incubated for 15 min at room temperature. The absorbance results were read at 450 nm.

### qRT-PCR

Total RNA form esophageal cancer patients and cells were extracted with TRIzol reagent (Invitrogen, Shanghai, China). We used ReverTra Ace (Toyobo Co., Ltd., Osaka, Japan) to reverse transcribe RNA into cDNA. was carried out using Thunderbird SYBR qPCR Mix (Toyobo Co., Ltd., Osaka, Japan) and LightCycler 2.0 (Roche Molecular Systems, Inc., Pleasanton, CA, USA) were used to perform the PCR process. 2^−ΔCt^ method was used to calculate the relative expression. GADPH and U6 were used as the control.

**Table:** 

Gene	R-primer	F-primer
SNHG3	TACAGGCGTGTAGCACCACAC	CTGGGATTACAGCTGTGAGCC
GAPDH	GCCAAGGTCATCCATGACAAC	ACCACTGACACGTTGGCAGTG
miR-186-5p	AAGAATTCTCCTTTTGGGCT	GTGCGTGTCGTGGAGTCG

### Western blot

Protein samples were obtained from KY-SE150 and Eca-9706 cell lines. The extracted protein samples were then suspended with loading buffers and deployed on the SDS-PAGE. After that, they were transferred onto PVDF membranes. In the end the blotting was visualized by ECL. The primary antibody of METTL3 was purchased from Abcam.

### Cell proliferation ability

Cell viability was evaluated using a CCK‑8 assay. Cells were resuspended and plated in a 96‑well plate at the concentration of 1 × 10^3^. As for treatment group, platinum (IC50) was added to treatment group and incubated for 24 h. After cultured for 24, 48, 72, 96 and 120 h respectively, 10 µl CCK‑8 was added to each well and incubated for 2 h; PBS was used as the blank control. The absorbance was detected at 450 nm.

### Cell apoptosis

FAM-FLICA® Caspase-3/7 Assay Kit was used to detect the apoptosis rate. 1:35 FLICA was added and incubated for 1 h according to the manufacturer’s guidelines. The caspase3/7 activity was evaluated by the fluorescence microscope (FAM-FLICA excites at 492 nm and emits at 520 nm).

### Dual luciferase reporter gene assay

The pGL3-SHNG3 and pGL3-METTL3 promoter (Promega, MA, USA) was transfected with pcDNA3.1/MIR-186-5p and pcDNA3.1 vector or sh-MIR-186-5p and sh-NC into KY-SE150 cell lines. The wild-type (WT) and mutant (Mut) binding sites of SNHG3 or METTL3 sequence was cloned into pmirGLO luciferase vector (Promega) to construct SNHG3 or METTL3-Wt and SNHG3 or METTL3-Mut, which were then co-transfected respectively with miR-186-5p mimics or NC mimics into KY-SE150 cell line. The luciferase activity was detected using Dual-Luciferase Reporter Assay System (Promega).

### RNA pull-down assay

The miR-186-5p-WT, miR-186-5p-Mut and NC were synthesized and biotin labeled by Genecreate (Wuhan, China). Then target cells were incubated with streptavidin-coated magnetic beads with RNase free bovine serum albumin for 48 h. Pull-down assay was carried out in biotin-coupled RNA complex. qRT-PCR was used to detect the expression of SNHG3.

### Xenografts

We have recruited 10 nude mice who were supplied by Animal center of Xi’an Jiaotong University. We used 1 × 10^6^ SNHG3 knocked down KY-SE150 cells and NC lentivirus to inject into the right flank of mice to generate xenografts. Four weeks later, xenograft tumors were harvested for further analysis.

### Statistics

GraphPad Prism 8.2.1 was used for statistically analysis and data visualization (La Jolla, CA, USA). Student’s t test and one-way ANOVA were used to test the difference among different groups. P < 0.05 was considered as statistically significance. All experiments were repeated six times.

## Result

### SNHG3, miR-186-5p and m6A associated enzymes expression esophageal cancer

**Fig. 1:**
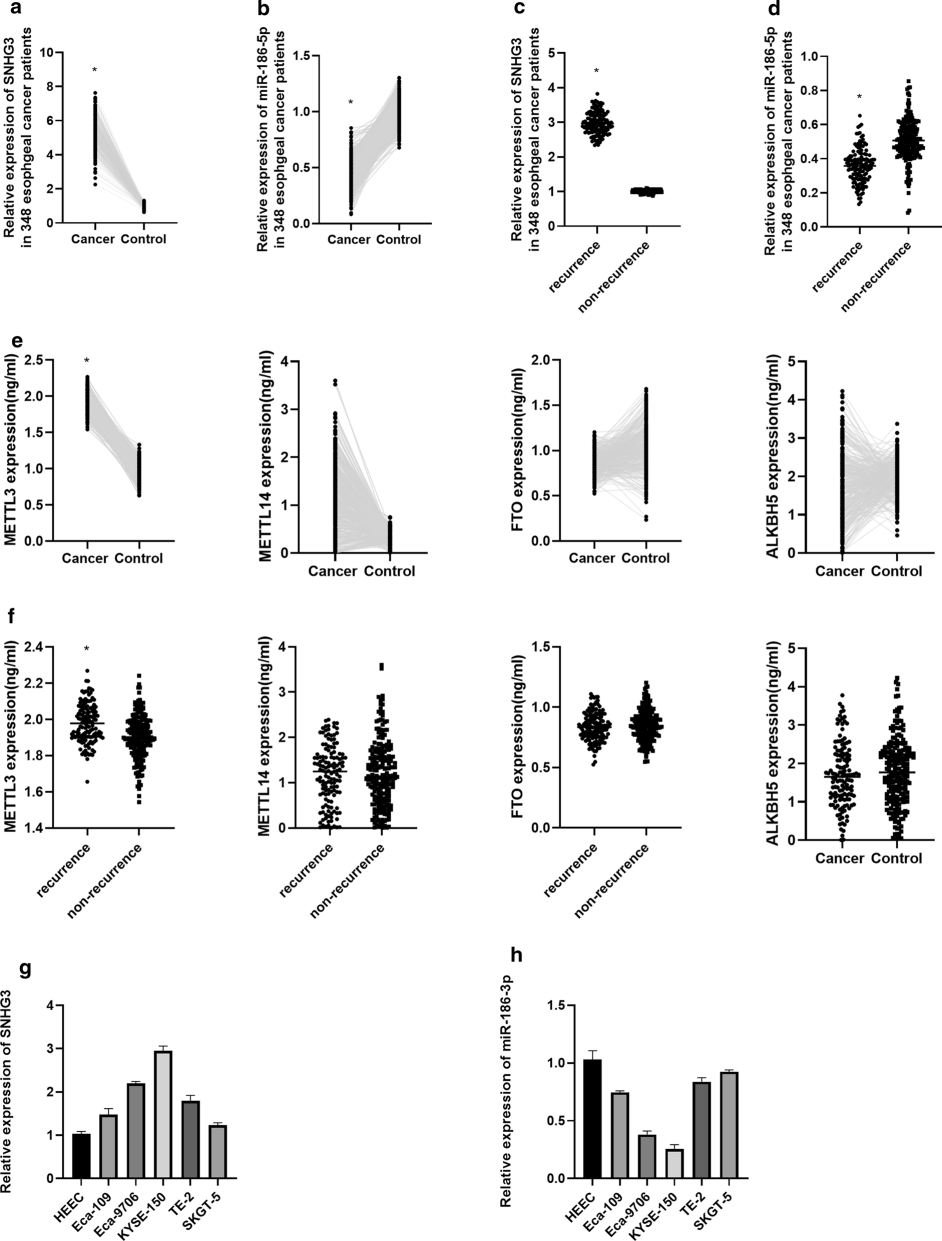
**a** Expression of SNHG3 in 348 esophageal cancer patients. **b** Expression of miR-186-5p in 348 esophageal cancer patients. **c** Expression of SNHG3 in recurrence and non-recurrence groups. **d** Expression of miR-186-5p in recurrence and non-recurrence groups. **e** Expression of METTL3, METTL14, FTO and ALKBH5 in 348 esophageal cancer patients. **f** Expression of METTL3, METTL14, FTO and ALKBH5 in recurrence and non-recurrence groups. **g** Expression of SNHG3 in esophageal cancer cells. **h** Expression of miR-186-5p in esophageal cancer cells

qRT-PCR showed that SNHG3 was highly expressed and miR-186-5p was lowly expressed in 348 esophageal cancer patients compared with their normal esophageal tissues; moreover, we noticed that SNHG3 expressed highly in recurrent esophageal cancer patients, however, miR-186-5p expressed lowly in recurrent esophageal cancer patients (Fig. 1a–d). Then, ELISA result showed that METTL3 and METTL14 were highly expressed in esophageal cancer, whereas FTO and ALKBH5 expressed lowly in esophageal cancer (Fig. 1e). According to Fig. 1f, we found that METTL3 expressed highly in recurrent esophageal cancer patients, however, METTL14, FTO and ALKBH5 were not differentially expressed between recurrent and non-recurrent esophageal cancer patients. After that, we have found that SNHG3 was highly expressed and miR-186-5p was lowly expressed in esophageal cancer in vitro (Fig. 1g, h).

### Cisplatin (CDDP), carboplatin (CPB) and oxaliplatin (L-OHP) could enhance the m6A level through up regulating SNHG3, miR-186-5p and METTL3

**Fig. 2:**
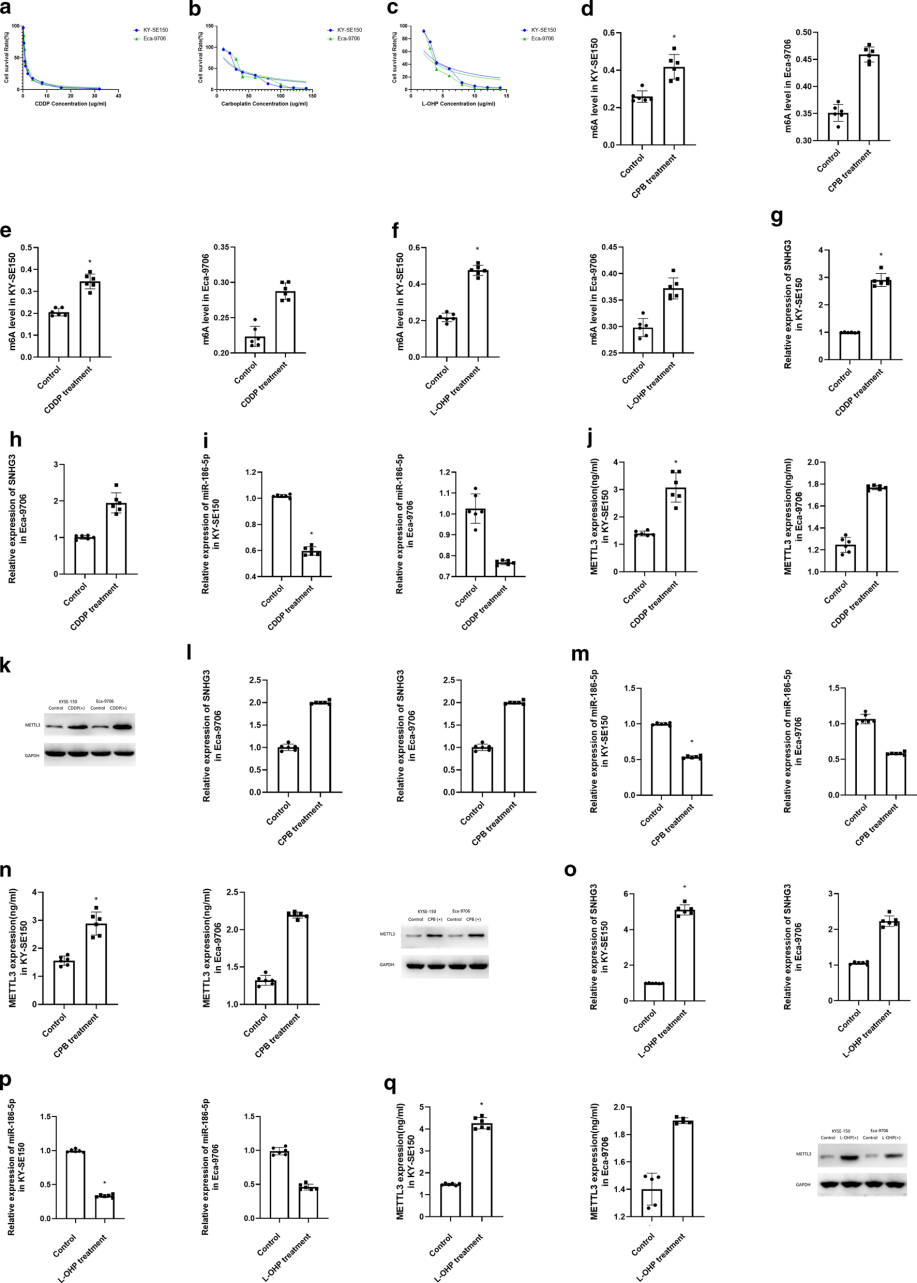
**a** IC50 for CDDP treated KY-SE150 and Eca-9706. **b** IC50 for CPB treated KY-SE150 and Eca-9706. **c** IC50 for L-OHP treated KY-SE150 and Eca-9706. **d**–**f** m6A level for CDDP, CPB and L-OHP treated LY-SE150 and Eca-9706. **g**–**i** SNHG3, METTL3 and miR-186-5p level for CDDP treated LY-SE150 and Eca-9706. **j**–**l** SNHG3, METTL3 and miR-186-5p level for CPB treated LY-SE150 and Eca-9706. **m**–**o** SNHG3, METTL3 and miR-186-5p level for L-OHP treated LY-SE150 and Eca-9706

The IC50 for CDDP is 0.76 ug/mL in KY-SE150 and 0.72 ug/mL in Eca-9706 (Fig. 2a). The IC50 for CPB is 29.53 ug/mL (KY-SE150) and 23.17 ug/mL (Eca-9706) (Fig. 2b). Besides, the IC50 for oxaliplatin is 3.19 ug/mL (KY-SE150) and 3.03 ug/mL (Eca-9706) (Fig. 2c). Then we treated KY-SE150 with IC50 for CDDP, CPB and L-OHP respectively for 24 h. After that, we found that CDDP, CPB and L-OHP could enhance the m6A level in KYSE-150 and Eca-9706 (Fig. 2d–f).

In KY-SE150 and Eca-9706, after treated with CDDP, we have found increased expression of SNHG3 and METTL3, but miR-186-5p expression was inhibited (Fig. 2g–i). Besides, CPB and L-OHP induced the expression of SNHG3 and METTL3, and suppressed the expression of miR-186-5p as well (Fig. 2j–o).

### SNHG3 and miR-186-5p regulates the m6A level of esophageal cancer by targeting METTL3

**Fig. 3:**
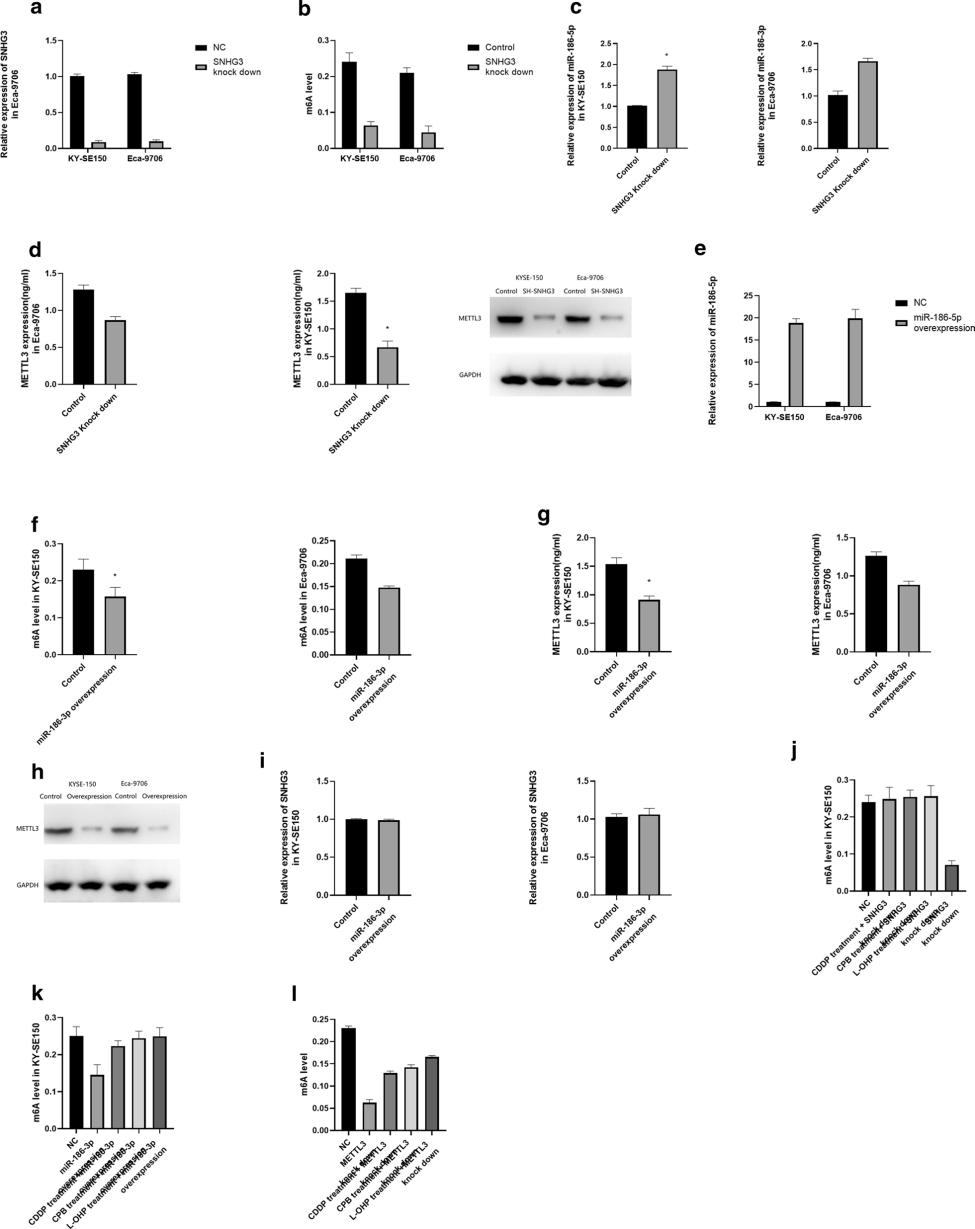
**a** Expression of SNHG3 in SNHG3 knocked down KY-SE150 and Eca-9706. **b** m6A level in SNHG3 knock down KY-SE150 and Eca-9706. **c** miR-186-5p expression in SNHG3 knock down KY-SE150 and Eca-9706. **d** METTL3 expression in SNHG3 knock down KY-SE150 and Eca-9706. **e** Expression of miR-186-5p in miR-186-5p overexpressed KY-SE150 and Eca-9706. **f** m6A level in miR-186-5p overexpressed KY-SE150 and Eca-9706. **g** METTL3 expression in miR-186-5p overexpressed KY-SE150 and Eca-9706. **h** SNHG3 expression in miR-186-5p overexpressed KY-SE150 and Eca-9706. **i** Rescue experiments for the effect of platinum treatment and SNHG3 knock down on m6A level. **j** Rescue experiments for the effect of platinum treatment and miR-186-5p overexpression on m6A level. **k** Rescue experiments for the effect of platinum treatment and METTL3 knock down on m6A level

We have successfully knocked down SNHG3 in both KY-SE150 and Eca-9706 (Fig. 3a).

After down-regulating SNHG3, we have found suppressed m6A level, down-regulated METTL3 and up-regulated miR-186-5p (Fig. 3b–d). Moreover, miR-186-5p overexpression resulted in suppressed m6A level and down-regulated METTL3 as well (Fig. 3e–g), however, the expression of SNHG3 was not significantly influenced (Fig. 3h). Then we treated SNHG3 down regulated KYSE-150 cell with CDDP, CPB and L-OHP, m6A level could be rescued by SNHG3 down regulated (Fig. 3i). Besides, increase in m6A level caused by platinum could be rescued by miR-186-5p overexpression and METTL3 knock down. (Fig. 3j, k). Therefore, we hypothesized that SNHG3 and miR-186-5p could regulate m6A level by targeting METTL3.

### SNHG3 promotes esophageal cancer proliferation and inhibits apoptosis by targeting miR-186-5p

**Fig. 4:**
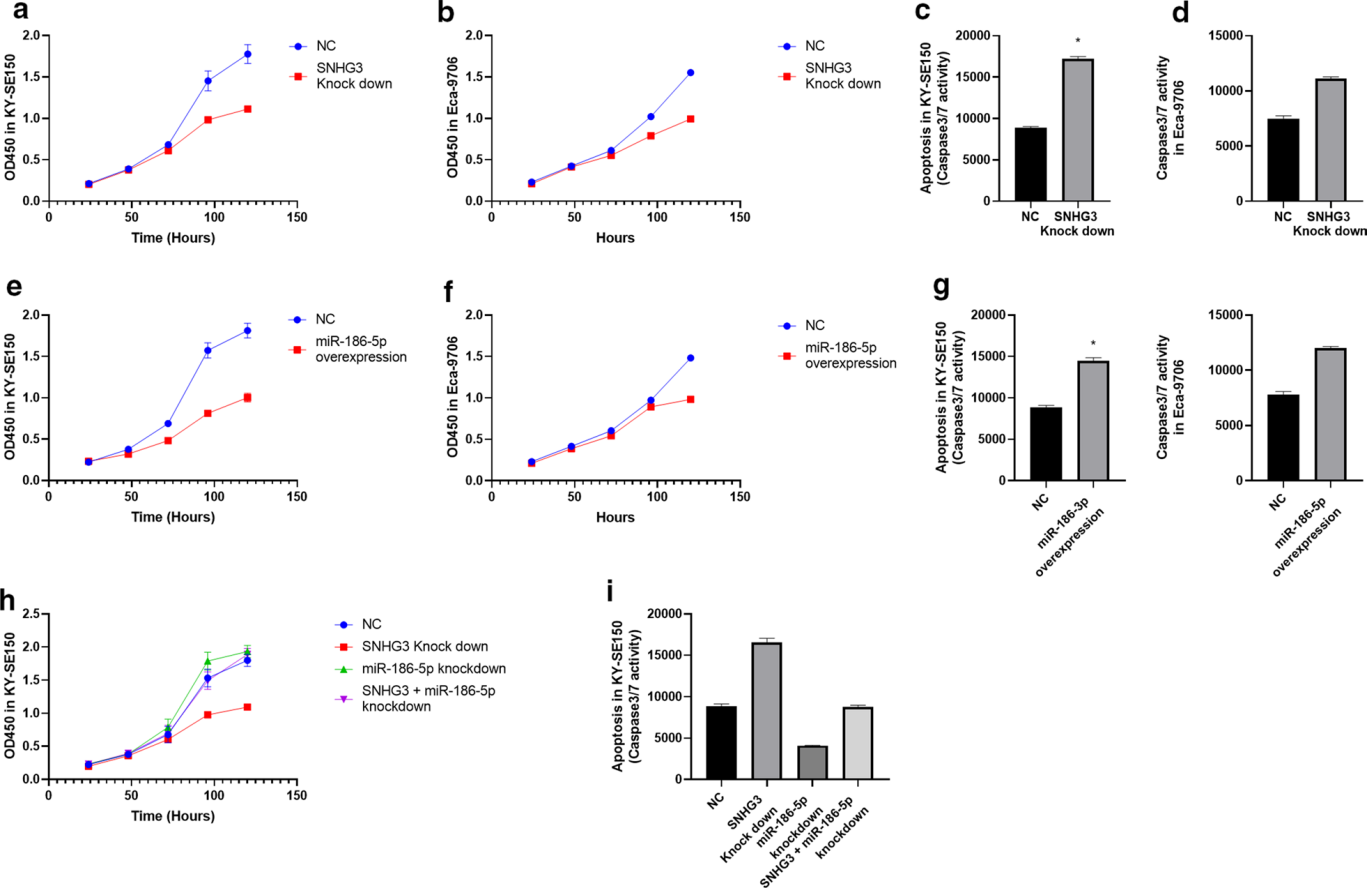
**a**, **b** Cell proliferation rate for SNHG3 knock down in KY-SE150 and Eca-9706. **c**, **d** Apoptosis rate for SNHG3 knock down in KY-SE150 and Eca-9706. **e**, **f** Cell proliferation rate for miR-186-5p overexpression in KY-SE150 and Eca-9706. **g** Apoptosis rate for miR-186-5p overexpression in KY-SE150 and Eca-9706. **h** Rescue experiments for the effect of SNHG3 knock down and miR-186-5p knock down on cell proliferation rate. **i** Rescue experiments for the effect of L-OHP treatment and SNHG3 knock down on apoptosis rate

In KYSE-150 and Eca-9706, we knocked down SNHG3 and found decreased proliferation rate and increased apoptosis rate (Fig. 4a–d). Besides, miR-186-5p overexpression led to decreased proliferation rate and increased apoptosis rate as well (Fig. 4e–g). Then, rescue experiments showed that the proliferation and apoptosis rate is not significantly different between miR-186-5p knock down plus SNHG3 knock down group and control group (Fig. 4h, i).

### SNHG3 directly interacts with miR-186-5p

**Fig. 5:**
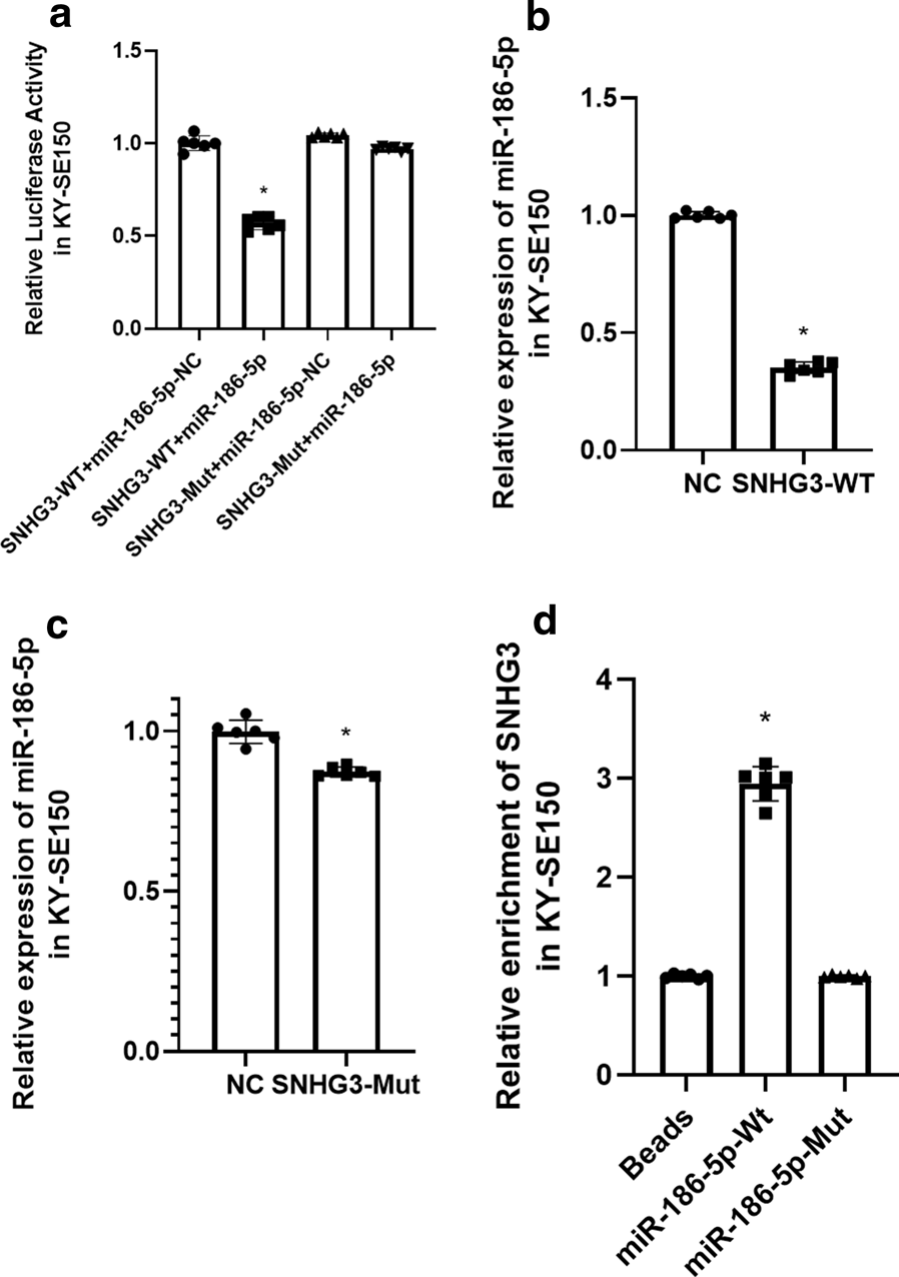
**a** Dual luciferase activity for SNHG3 and miR-186-5p. **b** miR-186-5p expression in SNHG3-WT overexpression KY-SE150. **c** miR-186-5p expression in SNHG3-Mut overexpression KY-SE150. **d** RNA pull down for SNHG3 and miR-186-5p

Previously, we have shown that SNHG3 knock down could lead to overexpression of miR-186-5p (Fig. 3c). Then Dual luciferase Reporter gene assay showed that relative luciferase activity was significantly lower in SNHG3-WT group than that in SNHG3-Mut group (Fig. 5a; Supplementary Figure1). We then transfected SNHG3-WT and SNHG3-Mut in KYSE-150 respectively and found that miR-186-5p was significantly suppressed in SNHG3-WT group but not significantly influenced in SNHG3-Mut group (Fig. 5b, c). Furthermore, we have performed RNA-pull down assay and found that SNHG3 was significantly enriched in miR-186-5p-WT group (Fig. 5d). Therefore, we assumed that SNHG3 directly interacts with miR-186-5p.

### miR-186-5p binds to the 3’UTR of METTL3 to inhibit its expression

**Fig. 6:**
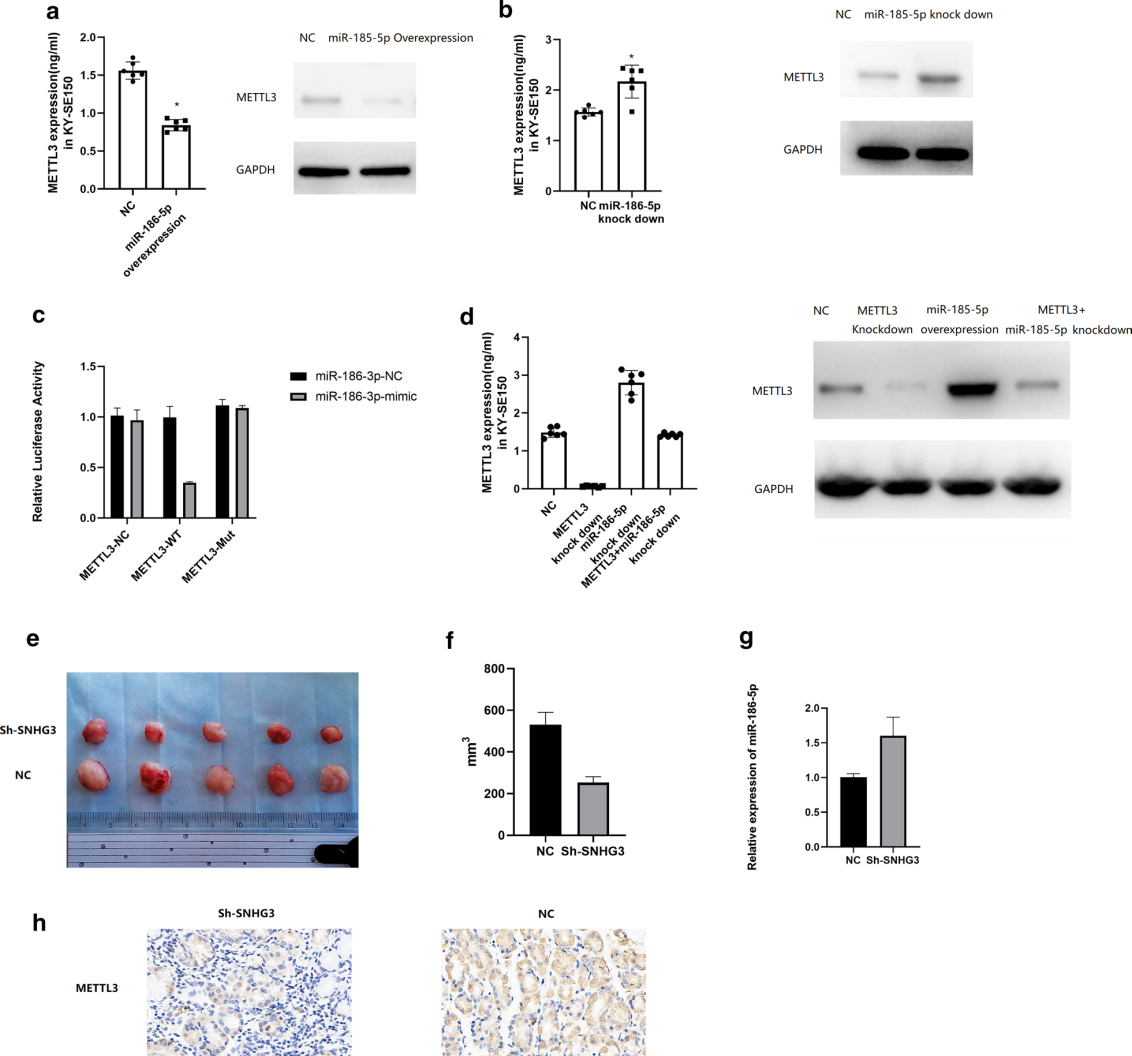
**a** METTL3 expression in miR-186-5p overexpressed KY-SE150 and Eca-9706. **b** METTL3 expression in miR-186-5p knocked down KY-SE150 and Eca-9706. **c** Dual luciferase activity for METTL3 and miR-186-5p in KY-SE150. **d** Rescue experiments for the effect of METTL3 knock down and miR-186-5p knock down on METTL3 expression in KY-SE150. **e**, **f** Xenografts for SNHG3 knocked and NC group. **g** Expression of miR-186-5p in SNHG3 knocked down xenografts. 6H Expression of METTL3 in SNHG3 knocked down xenografts

We found that miR-186-5p overexpression resulted in inhibited expression of METTL3, however, miR-186-5p knock down led to overexpression of METTL3 (Fig. 6a, b). Further dual luciferase reporter gene assay showed that relative luciferase activity was significantly suppressed in METTL3-WT group than that in METTL3-Mut group (Fig. 6c; Supplementary Figure 2). Then we have knocked miR-186-5p and METTL3 in the meantime, and found that METTL3 expression was not significantly different from that in control group (Fig. 6d). These results indicated that SNHG3 could interact with miR-186-5p to regulate the expression of METTL3.

After that, we noticed that SNHG3 knock down could decrease the xenografts growth in vivo (Fig. 6e, f). Then we harvested the xenografts and found aberrant high expression of miR-186-5p and low expression of METTL3 in SNHG3 knocked down xenografts (Fig. 6g, h).

## Discussion

It was reported that m6A methylation took part in various biological functions, including chemotherapy resistance, by modifying target RNAs [7]. It is found 0.1%-0.4% of adenosines from isolated RNA are modified by m6A, which accounts for 50% of total methylated ribonucleotides [16]. Previous studies have demonstrated that m6A methylation affects the physiological processes, including DNA damage repair, embryogenesis, heat shock responses, metastasis and proliferation [17–20]. In this manuscript, we found that platinum (CDDP, CPB and L-OHP) significantly induced the m6A level in KY-SE150 and Eca-9706 cell lines. Therefore, we assume that m6A regulation might be a novel way to control platinum resistance.

Furthermore, our study indicated that although METTL3, METTL14, FTO and ALKHB5 differentially expressed in esophageal cancer patients’ tissues and normal esophageal tissues, only METTL3 expression was related to esophageal cancer recurrence. It is reasonable to hypothesize that METTL3 might be key platinum resistance gene. In previous study, Taketo et.al showed that METTL3 knockdown sensitized pancreatic cancer to multiple anti-cancer reagents, including gemcitabine, 5-fluorouracil, cisplatin and irradiation as well [21]. In esophageal carcinoma, FTO [a demethylates N(6)-methyladenosine (m6A) RNA] and ALKBH5 were risk factors for poor prognoses as well [21]. It was shown that ALKBH5 knockdown could suppress proliferation and migration of ESCC cells [22]. Yang et al. found that knockdown of FTO could sensitize tumors to anti-PD-1 treatment [23]. Yan et al. found that overexpression of FTO could lead to tyrosine kinase inhibitor resistance in leukemia cells [8].

Moreover, we have found aberrant high expression of SNHG3 and low expression of miR-186-5p in esophageal cancer both in vitro and in vivo. We assumed that SNHG3/miR-186-5p played important role in regulating esophageal cancer progression. More importantly, we found that SNHG3/miR-186-5p expression was associated with m6A level in esophageal cancer. Further mechanism study showed that SNHG3 directly interacted with miR-186-5p to regulate the expression of METTL3. SNHG3 has been found to be necessary for cell growth: SNHG3 is vital for keeping embryonic stem cells’ self-renewal and pluripotency in embryonic stem cells [24]. Lu et al. stated that SNHG3 expression was higher in highly metastatic HCC cells (HCCLM3) than lowly metastatic HCC cells, such as Hep3B and PLC/PRF/5.Besides, SNHG3 also plays important roles in regulating drug resistance: high SNHG3 expression leads to poor survival and sorafenib resistance in hepatocellular carcinoma [25]. Fei et al. reported that in glioma SNHG3 overexpression could promote the cells proliferation, inhibit apoptosis rates, and accelerate the cell cycle progress by recruiting enhancer of zeste homolog 2 to the promoter of KLF2 and p21 [26]. Previous studies have indicated that SNHG3 could acted as ceRNA to regulate cancer biological process through binding microRNA [27]. For example, SNHG3 could promote laryngeal carcinoma proliferation and migration by binding miR-384 [27]. In osteosarcoma, SNHG3 could promote the progression of SNHG3 by absorbing miRNA-151a-3p and then upregulating RAB22A expression [28]. In this study, we assumed that SNHG3 could regulate the expression of METTL3 by sponging miR-186-5p. With the help of Dual Luciferase Reporter gene assay and RNA pull down assay, we found that SNHG3 could directly interact with miR-186-5p, which could bind to METTL3 to suppress its expression.

## Conclusion

We found that overall m6A level of esophageal cancer can be increased by platinum treatment, which would be an essential and important aspect for clarifying the molecular mechanism for platinum resistance. Furthermore, SNHG3/miR-186-5p, which can be induced by platinum, played an important part in involved in regulating overall m6A level by targeting METTL3 (Additional files 1, 2).

## Supplementary Information

**Additional file 1.**Figure S1: Luciferaseactivity for SNHG3 and miR-196-3p

**Additional file 2.**Figure S2: Luciferase activity for METTL3 and miR-196-3p

## Data Availability

Data and materials would be made available on request.

## Abbreviations

CDDP: Cisplatin
CPB: Carboplatin
L-OHP: Oxaliplatin
m6A: N6-methyladenosine
ESCC: Esophageal squamous cell carcinoma
