# Supplementary figures and images for: Targeting SNHG3/miR-186-5p reverses the increased m6A level caused by platinum treatment through regulating METTL3 in esophageal cancer
Source: Cancer Cell Int. 2021 Feb 17;21:114. doi: 10.1186/s12935-021-01747-9 (PMC7887820; doi:10.1186/s12935-021-01747-9)

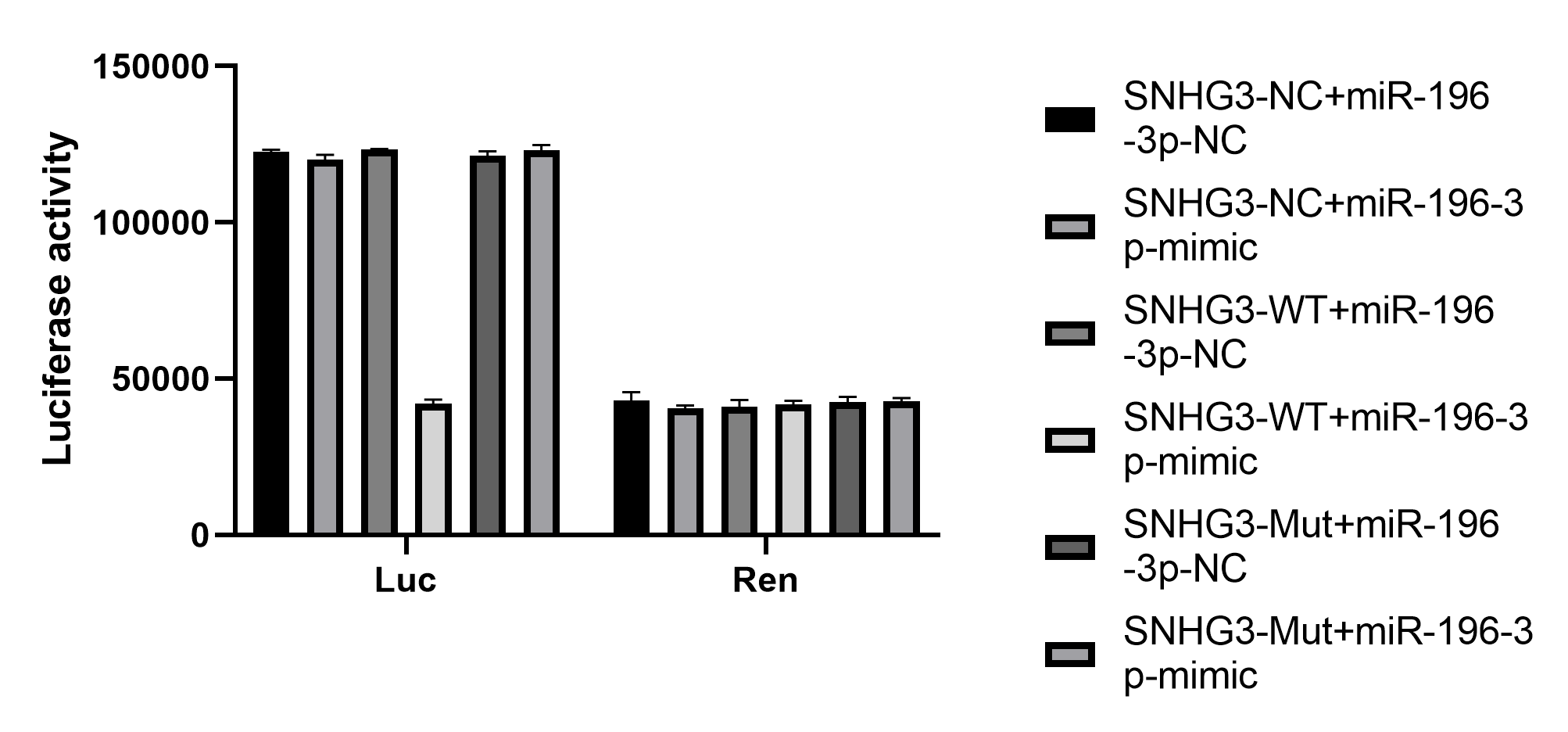

Supplement: Supplementary file 1 — Additional file 1.Figure S1: Luciferaseactivity for SNHG3 and miR-196-3p [file 12935_2021_1747_MOESM1_ESM.tif]

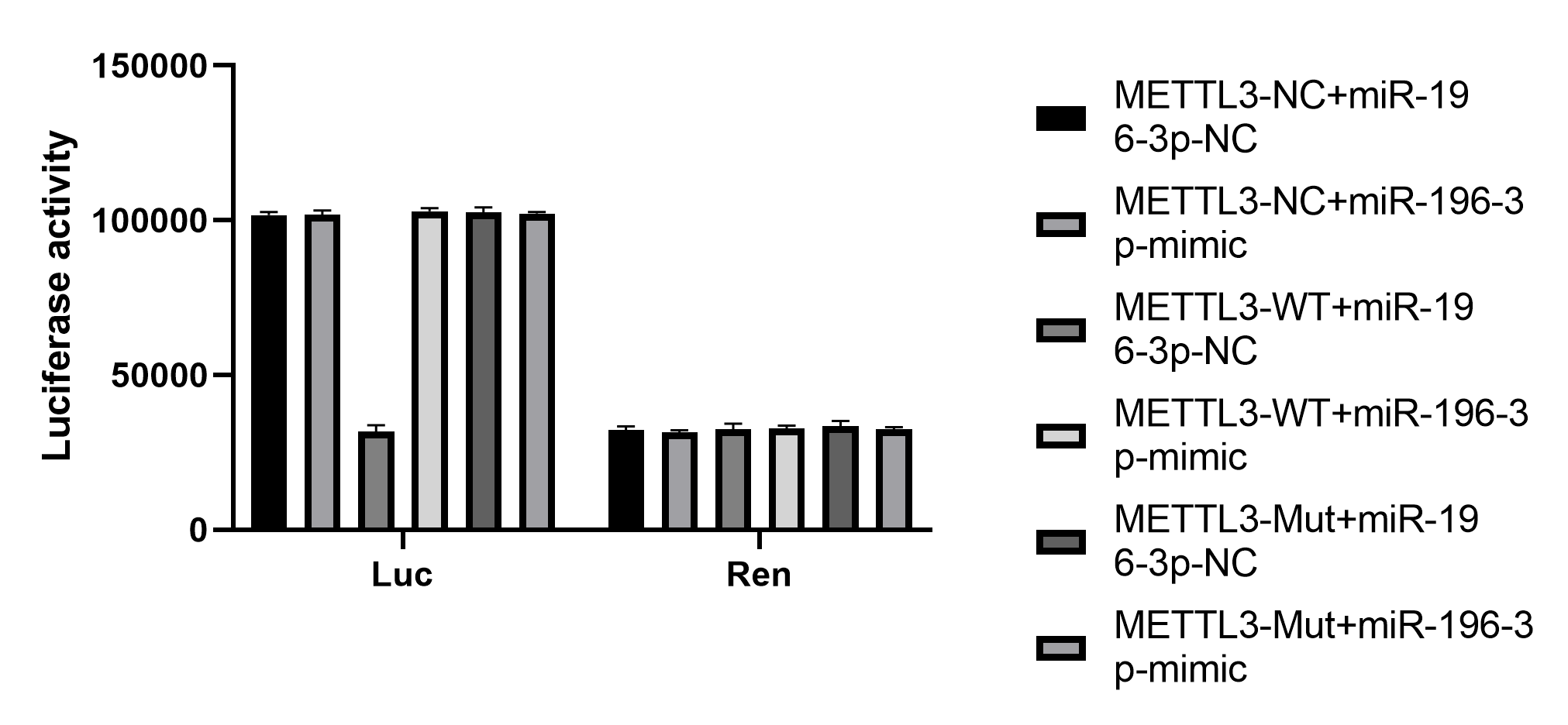

Supplement: Supplementary file 2 — Additional file 2.Figure S2: Luciferase activity for METTL3 and miR-196-3p [file 12935_2021_1747_MOESM2_ESM.tif]
